# Assessment of Subjective Well-Being in a Cohort of University Students and Staff Members: Association with Physical Activity and Outdoor Leisure Time during the COVID-19 Pandemic

**DOI:** 10.3390/ijerph19084787

**Published:** 2022-04-14

**Authors:** Stefano Quarta, Annalisa Levante, María-Teresa García-Conesa, Flavia Lecciso, Egeria Scoditti, Maria Annunziata Carluccio, Nadia Calabriso, Fabrizio Damiano, Giuseppe Santarpino, Tiziano Verri, Paula Pinto, Luisa Siculella, Marika Massaro

**Affiliations:** 1 Department of Biological and Environmental Sciences and Technologies (DISTEBA), University of Salento, 73100 Lecce, Italy; stefano.quarta3@unisalento.it (S.Q.); fabrizio.damiano@unisalento.it (F.D.); tiziano.verri@unisalento.it (T.V.); 2 Department of History, Society, and Human Studies, University of Salento, Via di Valesio, 73100 Lecce, Italy; annalisa.levante@unisalento.it (A.L.); flavia.lecciso@unisalento.it (F.L.); 3 Laboratory of Applied Psychology, Department of History, Society, and Human Studies, University of Salento, 73100 Lecce, Italy; 4 Research Group on Quality, Safety and Bioactivity of Plant Foods, Centro de Edafología y Biología Aplicada 40 del Segura-Consejo Superior de Investigaciones Científicas (CEBAS-CSIC), Campus de Espinardo, P.O. Box 164, 30100 Murcia, Spain; mtconesa@cebas.csic.es; 5 Institute of Clinical Physiology (IFC), National Research Council (CNR), 73100 Lecce, Italy; egeria.scoditti@ifc.cnr.it (E.S.); maria.carluccio@ifc.cnr.it (M.A.C.); nadia.calabriso@ifc.cnr.it (N.C.); 6 Cardiovascular Center, Paracelsus Medical University, 90471 Nuremberg, Germany; gsantarpino@gvmnet.it; 7 GVM Care and Research, Città di Lecce Hospital, 73100 Lecce, Italy; 8 Cardiac Surgery Unit, Department of Experimental and Clinical Medicine, University “Magna Graecia”, 88100 Catanzaro, Italy; 9 Instituto Politécnico de Santarém, Escola Superior Agraria, 2001-904 Santarem, Portugal; paula.pinto@esa.ipsantarem.pt; 10 Life Quality Research Centre (CIEQV), IPSantarém/IPLeiria, 2040-413 Rio Maior, Portugal

**Keywords:** subjective well-being (SWB), distress, mental health, general health, quality of life, social relationship, sport practice, time spent in nature

## Abstract

Time spent outdoors and physical activity (PA) promote mental health. To confirm this relationship in the aftermath of COVID-19 lockdowns, we explored individual levels of anxiety, depression, stress and subjective well-being (SWB) in a cohort of academic students and staff members and tested their association with sport practice, PA at leisure time and time spent outdoors. Our cross-sectional study collected data during the COVID-19 outbreak (April–May 2021) on 939 students and on 238 employees, who completed an online survey on sociodemographic and lifestyle features, depression, anxiety, stress, and SWB. Results showed that the students exhibited higher levels of anxiety, depression, and stress, and lower levels of SWB (*p* < 0.001 for all domains) compared to the staff members. Correlation analysis confirmed that PA and time spent in nature were associated to high mental health scores among staff and, more consistently, among students. Finally, mediation analyses indicated that the time spent in nature, social relationships, and levels of energy play a mediator role in the relationship between sport practice and SWB. Our evidence reinforces the protective role of time spent in nature in improving mental health, and provides support for policymakers to make appropriate choices for a better management of COVID-19 pandemic consequences.

## 1. Introduction

In recent years, there has been an increase in mental illness, which has had a surge as a result of the COVID-19 pandemic [1]. According to the World Health Organization (WHO), one in four people worldwide was affected by a mental disorder [1]. Among them, depression, anxiety, and stress are the most diffused in the general population [2,3]. According to the tripartite model of psychopathology [4], a high-order dimension, termed distress, includes the three above-mentioned dimensions. The other side of distress is psychological well-being, conceptualized as a contribution of the presence of positive affective states (e.g., vitality, energy, enthusiasm) and the absence of negative ones (e.g., tiredness, irritability, hostility) [5]. Recently, the Organization for Economic Co-operation and Development (OECD) has proposed a revision of the psychological well-being construct in terms of subjective well-being (SWB), which includes self-evaluation of several dimensions, such as global satisfaction, meaning, affect, as well as social and economic aspects of life [6]. Within the OECD framework, we have recently developed and applied a 9-item SWB instrument with two subdomains, that include both hedonic and eudemonic dimensions: one domain reflects satisfaction with one’s life and with oneself, and the other reflects perceptions of negative feelings, such as greater perceptions of stress, depression, and feeling unable to cope with daily tasks, along with items that capture perceptions of fatigue and body energy [7].

A strong negative relationship between distress and psychological well-being has been corroborated by several studies in the general population [8,9,10]. More specifically, a number of studies that have focused on the mental health of the academic students [11,12,13,14,15] have yielded worrying results, highlighting, on the one hand, a hyperbolic increase in the prevalence and severity of these psychological problems and, on the other, a decrease in the perception of the quality of life.

While physical activity (PA) has long been associated with a reduced risk of mortality and morbidity from chronic and degenerative diseases [16,17,18,19,20,21], the research interest on the role of PA on mental health has emerged more recently. PA has been related to an improvement in general well-being [22], enhanced perception of quality of life [23,24] and mood, as well as to a significant reduction in anxious and depressive symptomatology [25]. To explain such evidence many biological mechanisms have been suggested, including increased cerebral blood flow, and oxygen delivery to brain tissues, reduction in muscle tension, and increase in the serum concentrations of endocannabinoid receptors and satisfying neurotransmitters like serotonin [26]. Correspondently, the lack or a reduced rate of PA have been related to an increased risk of emotional instability, anxiety, and depression [27]. However, the most consistent results linking PA to a better mental health have been obtained when assessing the effects of PA in more advanced age groups [28], while studies looking at PA in younger people have produced a mixed picture with some studies suggesting weak association [29], and others suggesting a more persistent association between PA and mental health outcomes [30]. This lack of consistent evidence has been partly attributed to the use of measuring instruments that, when applied in isolation, do not comprehensively assess all aspects of mental health. This suggests the usefulness of applying a combination of tools to achieve a better evaluation of the mental health perception [31]. Furthermore, since PA has been conceptualized as a “complex multidimensional practice”, the evaluation of the environmental context where PA is carried out might be important for a more complete understanding of its relationship with specific mental health outcomes [32]. In this context, a growing body of research shows that exercise in a natural environment and spending time outdoors in green spaces with natural vegetation and/or near the sea/lake/river have positive effects on mental and physical health [33,34]. In particular, spending time outdoors seems to increase well-being in adolescents [35] and to decrease depressive symptoms in adults [36]. Of note, compared with just exposure to green space, nature-based activities and practicing physical exercise outdoors ensures a higher well-being, and diminishes stress and anxiety [37,38]. Longer interaction with nature brings out more intense social support and engagement in purposeful activities, which have been hypothesized as possible mechanisms implicated in the observed health benefits [37].

The long-lasting COVID-19 outbreak has caused drastic changes in people’s lifestyle and daily behaviors and routines. A number of factors related with this new situation (e.g., overload of confusing information about the COVID-19 infection, its vaccination and treatment, difficulties in the access to the healthcare system, the uncertainty regarding the financial scenario, etc.) have triggered an increase in feelings of fear, anxiety, worries, depression, and stress symptoms with a considerable negative impact on mental health [39,40]. In addition, in Italy, the long-term confinement, and the time spent working and/or studying from home have dramatically impaired social relationships as well as reduced the time spent outdoors [41] and the time practicing PA [42,43]. Overall, the COVID-19 pandemic has worsened both physical and mental health. This was particularly true for younger people, including adolescents and early adults who both featured with very active social habits based on multiple social relationships [44,45]. Since mental disorders, if undiagnosed and/or untreated, can become chronic and their severity increase later in life [46], new and better designed programs to protect and promote mental health among young people are needed [47]. To this aim, we further require an updated understanding of what young people have experienced during the COVID-19 pandemic, in terms of mental health and lifestyle habits most affected by home confinement and social distancing including physical and outdoor activities.

With this background in mind, the main objectives of this study are:

1. the simultaneous use of multiple complementary measurement tools to comparatively assess the mental health perceptions of an Italian subpopulation of young university students versus a subpopulation of adult university employees during a specific period of the pandemic COVID-19;
2. the assessment of the levels of PA, leisure activity and perception of energy levels in the two subpopulations during the same period;
3. evaluating the association between (1) and (2) through correlation analyses and a mediation model, which combine the role of time spent in nature, perceived individual energy level, and social relationships on the relationship between PA and subjective well-being.

A better understanding of the relationship between PA and mental health can both support the clinical practice of psychologists and/or psychiatrists and help policymakers design new, more comprehensive guidelines to promote mental health in the population.

## 2. Materials and Methods

### 2.1. Study Design and Ethics

Due to the exceptionally rapid spread of the COVID-19 virus, to ensure the safety of residents, the Italian government imposed a strict nationwide lockdown starting 10 March 2020. As a result, non-essential commercial and industrial activities were closed, sporting and cultural events and religious activities were suspended, and a strict closure of schools and universities was imposed throughout the entire country. University students were compelled to undertake distance learning until the end of September. Subsequently, further restrictive measures were introduced due to a new “wave” of COVID-19 that began in November 2020. This time, Italy was divided into three “coloured” zones (red, orange, yellow), with different restrictive measures depending on the severity of the spread of COVID-19 at the regional level. In Puglia, where the University of Salento is located, the closure of schools and universities was ordered until the end of March due to the higher severity of the pandemic. Access to courses and exams was again only allowed through remote platforms. Administrators and teachers were allowed to work from home until the end of the outbreak period. Within this framework, data were collected via a cross-sectional online survey between April and May 2021, a period immediately following the second lockdown of the COVID-19 outbreak in Italy.

The University of Salento Ethical Committee approved the research (No. 0056300). Predefined inclusion criteria were: (1) adults ≥ 18 years old of both sexes; (2) currently living in Italy; and (3) being an academic student or a University staff member. No selection criterion was applied in terms of educational level, health condition, social, and cultural background. Students and employees were invited to fill a Google online questionnaire through institutional communications. Participants read the information sheet and electronically signed the consent form before completing the questionnaire. Participation was voluntary, and unpaid. Data were collected anonymously following the Google security protocols.

### 2.2. Procedure and Measures

The survey took 15 to 20 min to be completed and included the following different sections:

1. *Socio-demographic characteristics* (age, sex, nationality, marital status, cohousing conditions, total household income per month, employment status);
2. *Body health status* (based on the reported weight and height by the participants and the estimation of the body mass index (BMI), as well as by the self-reported declaration of any recent diagnosed disease (yes/no);
3. *Comprehensive evaluation of the mental health perception*. We applied three independent psychological tools.

The Depression Anxiety Stress Scales Short Version (DASS-21) [48] consists of 21 self-reported items that measure three dimensions: anxiety (items 2, 4, 7, 9, 15, 19 e 20), depression (items: 3, 5, 10, 13, 16, 17 e 21) and stress (items 1, 6, 8, 11, 12, 14 e 18). The higher-order distress is calculated as the mean value of the three dimensions. In the present study, the questionnaire was administered with items exploring the levels of distress considering the previous 7 days. All items were evaluated on a 4-point Likert scale: 0 (Did not apply to me at all), 1 (Applied to me to some degree, or some of the time), 2 (Applied to me to a considerable degree or a good part of time), and three (Applied to me very much or most of the time). Partial scores were calculated as the mean values of items in each domains and ranged from 0–3, with higher scores indicating higher depression, anxiety, stress and distress levels. In the present study, the DASS-21 depression, anxiety and stress scores were also categorized as ‘normal’, ‘mild’, ‘moderate’, ‘severe’, and ‘extremely severe’ as indicated in DASS-21 manual instructions [48].

The WHOQoL-Brief questionnaire is an instrument that measures global quality of life [49]. This instrument consists of a series of questions originally divided into four domains: (I) physical health; (II) psychological health; (III) social relationships; and (IV) environmental quality of life, and two additional items on general perceptions of quality of life and satisfaction with health, rated on a 5-point Likert scale ranged from 1 (not at all) to 5 (extreme amount). In our survey, we included items belonging to the psychological health domain including: (I) *How much do you enjoy your life?*; (II) *To what extent do you feel your life is meaningful?*; (III) *How well can you concentrate?*; (IV) *Can you accept your physical appearance?*; (V) *How satisfied are you with yourself?*; (VI) *How often do you have negative feelings such as bad mood, despair, anxiety, depression?*. Furthermore, we included items related to the quality of social relationships such as (I) *How satisfied are you with your personal relationships?*; (II) *How satisfied are you with your sex life?*; (III) *How satisfied are you with the support you receive from your friends?*; alongside a preliminary question assessing the perceived general health as (I) *Are you satisfied with your health?*. The higher the score, the higher the quality of life. All subscale values were calculated as an average value.

The 9-item SWB instrument (9-item SWB index) consists of nine questions selected from previously validated instruments according the OECD recommendations [50] and that specifically address the hedonic and eudemonic dimensions of psychological well-being. This index has been previously shown to be a reliable tool, with adequate internal consistency, composite reliability, and convergent validity [7]. The instrument is constituted by two subdomains: a positive affect (C1) and a negative affect (C2) subdomains [7]. Briefly, (I) five items were adapted from the OECD core questions [50] and included: Life satisfaction: *How satisfied are you with your life as a whole these days?*; Worthwhile life: *To what extent do you feel that the things you do in life are worthwhile?*; Feeling happiness: *How happy did you feel during the last week?*; Feeling worried: *How worried did you feel during the last week?*; Feeling depressed: *Did you feel depressed during the last week?*; (II) two items adapted from the Cohen Stress Perception Scale [51]: Feeling nervous and stressed: *During last week, how often did you feel nervous and stressed?*; Unable to cope: *During last week, how often did you feel that you were unable to cope with all the thing you had to do?*; (III) two items adapted from the Lee’s Fatigue scale [52]: Energetic: *Last week, how energetic did you normally feel in the middle of the day?*; Efficient: *Last week, how efficient did you normally feel in the middle of the day?* All 9 questions were assessed using an 11-points Likert scale, ranged from 0 = not at all, to 10 = completely/all the time. Items related to negative feelings are reverse scored and the final total score was calculated as average of all items: the higher the score, the higher the nine-item SWB. The positive subdomain (C1) was derived averaging the scores of the following five positive feelings: life worthwhile; efficient; life satisfaction; energetic; and feeling happy; the negative subdomain (C2) was derived averaging the score of the following four negative feelings: feeling nervous and stressed; feeling worried; unable to cope; feeling depressed.

The specific items included in each tool are summarized in Table 1.

4. *Energy levels*. The evaluation of energy levels was computed on three items adapted from the Lee’s Fatigue scale [51]: (1) *Last week, how energetic did you normally feel in the middle of the day?;* (2) *Last week, how tired did you normally feel in the middle of the day?;* and (3) *Last week, how efficient did you normally feel in the middle of the day?.* Total score was calculated as the average of all three items (Table 1).
5. *Physical activity habits*. The evaluation of the levels of PA of the participants was conducted by administering the following ad hoc question: *If you practice sport, on average, last month, how frequently did you practice exercise?* The answer was given according to the 4-point Likert scale and pass through points 0 = Never; 1 = Occasionally, but not regularly; 2 = Regularly, less than 150 min per week; 3 = Regularly, 150 min or more per week. The cut-off points were: low (never or occasionally) and moderate/high (regularly).

Participants were also asked if they involved themselves in leisure activity and if they practice PA within their leisure time. The ad hoc question administered was: *What type of activity do you practice more habitually at leisure time?* The answer was given, again, according to the 3-point Likert scale and pass through the following point: 0 = Activities that do not require physical activity (e.g., reading, watching TV), 1 = Relaxing activities (e.g., walking, gardening, slow biking) some times per week, 2 = Practice sport or intense physical activities. The cut-off points were: low (if they do not practice PA at all or practice relaxing activities) and moderate/high (if they practice sport or intense physical activities). Finally, recreational nature contact or time spent in natural environments in the last month was addressed by asking participants: *During the last month, how often did you spend time in nature?* The answer was given always according to a 5-point Likert scale that passed through the following points: 0 = Never; 1 = Occasionally; 2 = Sometimes; 3 = Frequently; 4 = Almost all the time. The cut-off points were: low (if they never or just sometimes spent time in nature in a month) and moderate/high (if they frequently spent time in nature).

### 2.3. Covariates

As potential covariates requiring adjustment we included: age, sex, marital status, total household monthly income, smoking, night sleeping time, body health status and social relationships. The subpopulations (students vs. employees) were further added as a covariate. Age was categorized as follows: 18–24 years, 25–34 years, 35–44 years, 45–54 years, 55–64 years, >65 years. The categories for marital status were “single” and “married or in an analogous relationship”. The household monthly income was classified into six categories: (1) <EUR 500; (2) EUR 500–1000; (3) EUR 1000–1500; (4) EUR 1500–2000; (5) EUR 2000–2500; (6) =>EUR 2500.

### 2.4. Data Analysis

Statistical analyses were all performed with the Statistical Package for the Social Sciences (SPSS) statistical package for Windows (SPSS, Inc., Chicago, IL, USA). Sociodemographic, BMI, and mental health perception data are presented as % for ordinal or nominal variables, and as median and interquartile range (IQR) for scale variables. To facilitate comparison with other studies, we also present mean values and standard deviation (SD) for scale variables. Cronbach’s alpha was used to assess the reliability of the mental health tools. To examine differences between groups, we applied the following non-parametric tests: Mann–Whitney U tests for scale variables and Chi-square tests and Fisher’s exact test for ordinal and nominal variables (significant *p*-values < 0.05). To calculate the effect size the following formula was applied: r = z/√n where z is the standardized test statistic z value and n is the number of paired samples. An effect size (r) less than 0.3 is defined as a small effect, between 0.3 and 0.5 a medium effect, greater than 0.5 a large effect [53]. Pearson’s r correlation was carried out to evaluate the association between variables. Spearman’s ρ non-parametric partial correlation was carried out to evaluate the association between PA, time spent in nature and mental health scores adjusted with the previously described covariates. The intra-class correlation coefficient (ICC) test with a 95% confidence interval was applied to assess the reliability of the 9-item SWB score and its related positive and negative subdomains against the already validated DASS-21 subscales and WHOQoL psychological health domain [54].

**Figure 1:**
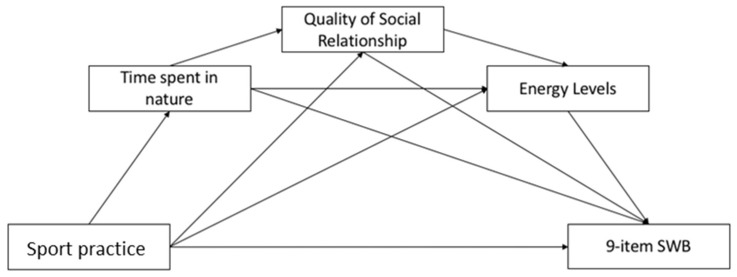
Hypothesized mediation model.

Mediation analysis was performed using PROCESS v3.0, applying the Model 6 and 5000 bootstraps inference for model coefficients. In the hypothesized mediation model (Figure 1), the predictor variable was the sport practice, the outcome was the 9-item SWB, and the mediators (Ms) were the time spent in nature (M1), the perceived quality of life in terms of social relationship dimension (M2) and the energy levels (M3). Sex, total household monthly income, marital status (with partner vs. without a partner), BMI range (underweight vs. normal weight vs. overweight vs. obese), diagnosed pathology (presence vs. absence), smoking (yes vs. no) and subpopulation (students vs. employees) were included as covariates.

## 3. Results

### 3.1. Participants Sociodemographic Characteristics and Body Health Status

The socio-demographic characteristics of the sample population are presented in Table 2. A total of 1177 individuals completed valid questionnaires. The total sample population included 939 students and 238 employees. All respondents were Italian and lived in the south of Italy (i.e., Apulia) at the time of the survey. From the analysis of the data distribution, significant differences emerged between the two subpopulations studied. Regarding sex, the participation of females was in general higher than that of males (female: 70.7%), especially among students, 75% of whom were female, while among employees the percentage of females was 54.2%. Most of the students were between 18 and 34 years old while most of the employees were between 45 and 64 years old. There were also significant differences in marital status, as most employees were married and had at least one child, while most students were single and only a small percentage (5.6%) had one child or more. The mean number of household members was higher for students than for staff (3.69 ± 0.99 and 2.92 ± 1.17, respectively, *p* < 0.0011). Family income was higher among employees than students, with 50% of employees having a monthly income > EUR 2500, while the majority of students reported a family income between EUR 1000 and 2000. Although most participants fell into the normal weight category, we found statistically significant differences for the BMI between students and employees (23.14 ± 4.21 Kg/m^2^ and 24.88 ± 4.00 Kg/m^2^, respectively, *p* < 0.0011). The percentage of overweight and obese individuals was higher among the staff members than among the students (42.1% and 29.4%, respectively). Finally, regarding the general health condition, 86.9% of students and 79.0% of employees were classified as healthy, as they did not report the diagnosis of any diseases.

### 3.2. Evaluation of Distress and Subjective Well-Being in the Student and Staff Member Subpopulations

In order to obtain an overview of the mental health perception of both students and staff members in the aftermath of lockdown, three independent measuring tools were administered: the DASS-21, the WHOQoL-Brief, and the 9-item SWB index.

**Figure 2:**
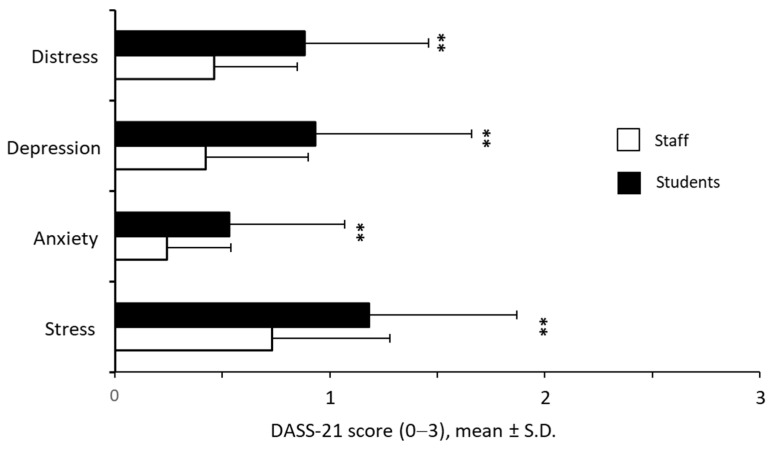
Comparison of the scoring for depression, anxiety, and stress perception as well as for the overall distress score between the two University subpopulations; ** *p* < 0.001.

Since one of the most efficient and widely used tools to assess symptoms of depression, anxiety and stress is the DASS-21 questionnaire [48], we decided to investigate the distress of students and staff members by applying this tool. As shown in Figure 2, the staff subpopulation displayed lower scores for depression, anxiety and stress perception (*p* < 0.001) than the students. The overall distress was ≈50% higher (*p* < 0.001) in students than in employees with mean values of 0.88 (±0.58) and 0.46 (±0.39), respectively. To assess the reliability of DASS-21 in the two sub-populations studied, we performed Cronbach’s alpha test for distress and the three subscales. Good to excellent values were observed for stress, anxiety and depression subscales both in students (α = 0.902 for stress, α = 0.822 for anxiety, α = 0.906 for depression) and staff (α = 0.888 for stress, α = 0.732 for anxiety, α = 0.875 for depression). Overall, excellent Cronbach’s alpha values were observed for distress with α = 0.942 in students and α = 0.927 in staff.

In Table 3 we additionally report the distribution of the DASS-21 scores as a function of depression, anxiety, and stress severity levels for the two subpopulations investigated. We observed that more than 50% of the students reported mild to extremely severe levels of depression and stress whereas ~40% showed mild to extremely severe levels of anxiety. In contrast, only ~20% of the staff members showed mild to extremely severe levels of depression and stress, and ~15% had mild to extremely severe levels of anxiety. Overall, these data corroborate that distress was worse among students than among employees.

**Figure 3:**
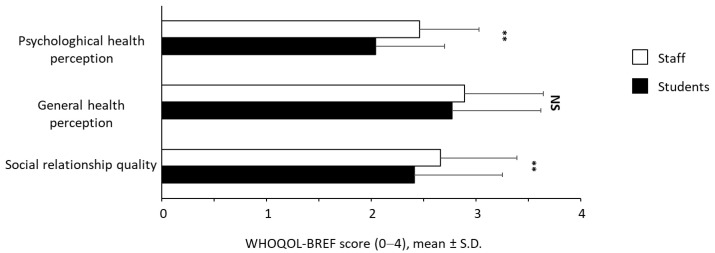
Quality of life scores assessed by WHOQoL-BRIEF questionnaire. N.S.: not significant; ** *p* < 0.001.

We next addressed the perceived psychological and general health, and the quality of social relationship by applying the WHOQoL-Brief questionnaire [55]. Results are shown in Figure 3. Staff members reported a significantly higher psychological health and better quality of social relationship compared to students. The perceived psychological health was ~20% higher in the staff members than in students (*p* < 0.001) with mean score values of 2.46 ± 0.57 and 2.04 ± 0.66, respectively. The difference between the two subpopulations regarding the quality of their social relationships was small yet significant (*p* < 0.001) with a mean value of 2.66 ± 0.73 for the staff participants, and 2.41 ± 0.84 for the students. In contrast, there was not a significant difference in terms of the perception of the general health condition (*p* = 0.059).

We additionally investigated SWB in the two subpopulations by implementing a recently developed tool, the 9-item SWB instrument, composed of a negative and a positive subdomain, that combine hedonic and eudemonic items [7].

**Figure 4:**
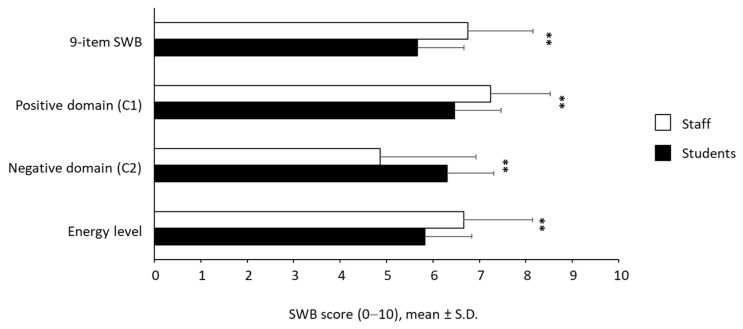
SWB scores (9-items SWB with its positive and negative domain scores and energy level). ** *p* < 0.001.

As shown in Figure 4, tightly aligned to the results obtained by DASS-21 and by WHOQoL-Brief questionnaires, staff members showed higher SWB scores than students with a *p*-value < 0.001 both for the global score and for the two sub-domains C1 and C2. We found that the 9-item SWB was higher in staff than students (6.75 ± 1.41 and 5.67 ± 1.55, respectively). Furthermore, staff showed higher mean values than students for the positive domain C1 (7.24 ± 1.28 and 6.47 ± 1.51, respectively) and, correspondingly, lower mean values than students for the negative domain C2 (4.86 ± 2.06 and 6.31 ± 2.07). In addition, participants were asked to answer some questions related to the perceived energy levels. As shown in Figure 4, staff subpopulation exhibited a statistically significant higher perception of the levels of energy than students (6.66 ± 1.48 vs 5.83 ± 1.63, *p* < 0.001). The reliability of 9-item SWB was excellent both in students (α = 0.873) and staff (α = 0.851). Furthermore, Cronbach’s alpha values were excellent also for positive (α = 0.873 in students and α = 0.851 in staff) and negative (α = 0.844 in students and α = 0.806 in staff) domains.

### 3.3. Validation of the 9-Item SWB Index with the Two Subdomains

In order to reinforce the applicability of the 9-item SWB index, as well as of its positive and negative subdomains, we performed a validation analysis, in both cohorts under investigation, using the DASS-21 and WHOQoL psychological health domain as comparative reference instruments. Results are shown in Table 4. Pearson correlation analyses between the 9-item SWB mean score and the WHOQoL psychological health domain mean score exhibited a strong positive correlation both for the staff subpopulation (r = 0.734, *p* < 0.001) and for the students (r = 0.801, *p* < 0.001). We additionally found a strong negative correlation with the DASS-21 distress mean score (r = −0.784 for the staff participants and r = −0.801 for the students, *p* < 0.001 in each group). We next evaluated the agreement between the 9-item SWB and the WHOQoL psychological health domain applying the ICC test. Again, the results showed a moderate, although still significant, agreement (*p* < 0.001) for both groups with a higher ICC value in students (0.713, 95%IC 0.673, 0.747) than staff (0.678, 95%IC 0.585, 0.751). The same analyses were performed to validate the two subdomains: the positive domain showed a strong positive correlation and a moderate yet significant agreement with the WHOQoL psychological health domain in each population considered with higher r and ICC values in students than staff. To validate the negative subdomain, we compared its value with the value obtained from DASS-21 subscales: the negative domain showed a strong positive correlation (*p* < 0.001) with distress and stress scores in both staff and students (r = 0.762; r = 0.740 for distress and r = 0.760; r = 0.745 for stress), yet a moderate, even still significant, correlation with depression and anxiety scores (*p* < 0.001). Similarly, for students, the ICC test also resulted in a moderate significant agreement (*p* < 0.001) with DASS-21 distress, depression and stress scores and a significant yet poor agreement (*p* < 0.001) with the DASS-21 anxiety score. Regarding the staff, there was a significant moderate agreement (*p* < 0.001) with the DASS-21 stress score and a significant poor agreement (*p* < 0.001) with all the other scores. Overall, we observed a moderate to strong correlation between the negative domain and DASS-21 distress and a poor to moderate agreement with all DASS-21 subscales.

### 3.4. Distribution of PA Levels among Students and Staff Members

In order to find out whether there is a relationship between practicing sport, PA and time spent in nature with mental health perception in the new social and economic scenario resulting from the succession of lockdowns, participants were asked to indicate the frequency of practicing sports, the type of activities performed in their free time, and the time spent in contact with nature.

As shown in Table 5, there were significant differences between the students and the staff members in terms of sports practicing and activities carried out in their leisure time (*p* < 0.001), whereas the differences in the time spent in contact with nature did not reach significance. During leisure time, ~66% of the students and staff members reported engaging in relaxing activities or activities that do not require physical effort, while ~33% of them engaged in sports activities. In terms of leisure time, employees preferred more relaxing activities than students did (44.1% and 33.1%, respectively), while students engaged in more sports (34.8% and 29.8%, respectively). In terms of sports activities, students are more active as more than 40% of staff members do not play sports, while ~40% of students play sports regularly. In terms of time spent in nature, there were small and statistically non-significant differences between the groups, with staff spending a little more time outdoors than students do.

### 3.5. Association between Distress, SWB, Physical Activities Levels and Time Spent in Nature

In order to explore the relationship between PA and mental health, in terms of distress and SWB, we dichotomized into two categories (low and moderate/high) the amount of sport practiced, the PA practiced in leisure time and the time spent in nature and divided participants accordingly. As shown in Table 6, those practicing more sport, more PA in leisure time and spent more time in nature reported lower scores for DASS-21 subscales and higher score for 9-item SWB. Notably, such relationships were higher and more consistently reproduced in students than in staff members. However, in the staff member cohort there was also evidence that the protective contribution of PA and nature determined better mental health conditions.

In order to confirm the association between sport practice, PA in leisure time and time spent in nature with better mental health condition we have performed non-parametric partial correlation analysis with all the indexes used. Results are shown in Table 7. As expected, after correcting for sex, age, pathology, marital status, family income, WHOQoL-Brief social relationship score, smoking and sleep habits, practicing of sport and PA at leisure time and time spent in nature significantly correlated with better scores for most of mental health indexes. Again, the strength of the correlations was higher and more reproducible in students than in staff members.

### 3.6. Mediation Models

Having shown that the 9-item SWB can accurately reflect measures of distress and psychological well-being (Table 4), we decided to use this shorter and flexible instrument in the following assessment.

**Figure 5:**
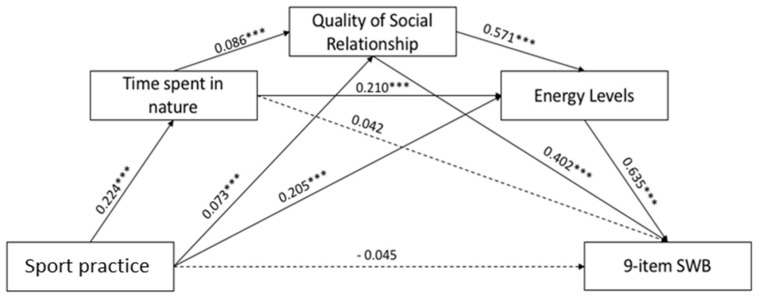
Results of the Mediation Model. Note: * *p* < 0.05; ** *p* < 0.01; *** *p* < 0.001. Only significant path coefficients are displayed; not significant paths were displayed by dotted lines.

Since the correlation analysis has shown that sport practice, time spent in nature, energy levels and mental health indexes were positively correlated with each other (Supplementary Table S1), we expected that the time spent outdoors, the improvement in energy level and the quality of social relationships could play a mediator(s) role in the relationship between the practice of sport and the SWB. In Figure 5, and in the corresponding Table 8, the obtained results are reported.

The direct path between sport practice and 9-item SWB was not significant whereas two significant indirect paths were found. Regarding the impact of the first mediator, i.e., time spent in nature, the indirect path on the 9-item SWB was not significant. Furthermore, results revealed a significant indirect path between sport practice and 9-item SWB via the mediation of the social relationship: the sport practice had an impact on the social relationship, which in turn had an impact on the 9-item SWB. Regarding the third mediation, findings showed a significant indirect path: sport practice had an impact on the energy levels which, in turn, had an impact on the 9-item SWB. With regard to the parallel mediation role of the time spent in nature, social relationship, and energy levels on the relationship between sport practice and 9-item SWB results showed a significant path. Overall, the mediation models showed the significant indirect path between the predictor variable and the outcome considering: (a) time spent in nature and social relationship; (b) time spent in nature and energy levels; (c) social relationship and energy levels. The total effect was β = 0.195 (*p* < 0.001).

## 4. Discussion

The worldwide monitoring and improvement of subjective well-being have been two of the main WHO goals since 2015 [56]. For the first time in the history of social policy, the attention was no longer focused on the maintenance and/or achievement of an acceptable physical health status, but was shifted to embrace also the maintenance or achievement of a good state of mental health. Unfortunately, these premises are no longer being met. Epidemiological analyses conducted over the past two years have invariably shown a worsening in psychological symptomatology and a general deterioration in mental health and global well-being, to which the spread of SARS-CoV-2 with its associated lockdowns and quarantines appears to have contributed significantly, especially and more severely in the younger age groups [57,58].

With this study, we aimed to assess SWB and distress in the later stages of the COVID-19 pandemic outbreak to confirm the upward trend in worsening perceptions of SWB, and to identify risk and protective factors for SWB in young adults compared with more mature individuals.

In terms of depressive symptom distribution, the most updated European report on mental health published prior to the spread of SARS-CoV-2, placed Italy in a good position with only 6% of Italian adults disclosing depressive symptoms [59,60] and 8.6% of Italian students disclosing psychiatric diagnoses in 2015 [61]. Such rates depicted a general good mental health condition when compared to our data now showing an increased level of depressive symptoms that were disclosed by 57% of students and 23% of staff. Our data provides support to previous studies on the same topic. The observed increased rates are in agreement with those reported by Ammar et al. [62] and Sibley et al. [63], that specifically compared mental health in pre- versus post-confinement conditions. Furthermore, the proportion of individuals in our survey who reported severe anxiety or depression symptoms is comparable to proportions observed in other studies in academic communities in France, Spain and Italy. In French and Spanish studies, the proportion reporting anxiety were 27% and 21% respectively, while the proportion reporting depression was 16% and 34% [64,65]. Similarly, in a multi-center survey on five academic communities in Italy, 20% of participants reported severe anxiety and depression symptoms with the most impacting factor recognized in being a student. In agreement with Brooks et al. [66], it is highly likely that the increased frequency of depressive and distress symptoms found in our samples could be causally linked to the COVID-19 outbreak. However, further studies are needed to confirm this association.

The vast majority of research on mental health, especially when carried out among academic students, investigates distress [15]. However, capturing distress highlights only one aspect of overall mental health, leaving an important gap in our understanding of perceived global health, quality of life and SWB. In particular, SWB is related to, but also distinct from, the absence of distress and mental illness, and much research is emphasizing its “resource function” for students and the general population [50]. Data gathered up to now on this kind of mental health assessment have been mostly of a correlational type, rather than experimental; however, recent longitudinal studies are furnishing a significant support to the role of SWB assessments. For example, from the analysis of data gathered over 28 years on 6856 individuals, after controlling for age, sex, education and baseline health, results show that SWB and positive feelings (but not negative feelings) significantly predicted lowered risks of all-, natural- and non-natural causes of mortality and were confirmed separately in younger (<55 in age) and/or older (>55) subsamples [67]. Similarly Boehm et al. based on a five-year follow-up of 7942 participants in the Whitehall II cohort, shows a consistent association between SWB and reduction in the risk of coronary heart disease [68]. Longitudinal studies addressing SWB among students have also highlighted promising results in terms of predictive power towards learning outcomes, academic performance and future work engagements [69,70,71]. This calls for more focus on SWB and appreciation of positive dimensions to promote population longevity and individual fulfillment. In our work, we measured global SWB and two related sub-indices, one capturing positive feelings and the other capturing negative feelings, in both students and staff, as previously reported by us [7]. In the paper by Andrade et al. [7], the SWB measurement was carefully entailed to allow for internationally reliable and valid assessment of mental health outcomes, while reducing questionnaire burden by choosing scales with low item numbers [7]. In addition to previous work, we have now validated the 9-item SWB index and both subdomains against reference indexes. Not surprisingly, we found high correlations between the comparative instrument scores of DASS-21 and WHOQoL psychological health domain with the 9-item SWB and associated subdomains (C1 and C2), thus, further enabling the use of these new shortened indexes in future surveys.

With respect to the data collected prior to the COVID-19 outbreak [7], we now observe a significant worsening in SWB scores (*p* < 0.001 at t-student test) and associated subdomains for both general (−3.4%, +15.4%, and −6.76% for the 9-item SWB, the negative, and the positive component, respectively) and student subsamples (−7.04, +21.34, −8.87 for the 9-item SWB, the negative, and the positive component, respectively), while scores for older age range subsamples remained unchanged, providing further evidence that the economic and social consequences of the COVID-19 outbreak have primarily affected younger and economically vulnerable populations [72].

The first evidence showing the positive impact of PA on mental health dates back to the 1980s and 1990s [73]. In particular, in 1992, the International Society of Sport Psychology endorsed the previous position sustained by the American National Institute of Mental Health, recognizing the link between regular exercise and mental health [74]. These consensus documents, for the first time, concluded and recognized that some psychological dysfunctions, including depression, anxiety, and stress could benefit from involvement in PA [74]. The evidence for a significant and positive relationship between PA and psychological outcomes was successively confirmed and recognized as even stronger in psychiatric individuals [75]. More recently, in order to improve cardiorespiratory, muscular and bone fitness, reduce the risk of NCDs and the risk of depression, the WHO has recommended, for adults aged between 18 and 64 years, to do at least 150 min of moderate-intensity aerobic PA or at least 75 min of vigorous-intensity aerobic PA throughout the week. The statement advices to include both recreational and leisure-time walking or cycling, household works, team or individual sport and planned exercise, in the context of daily, family, and community activities [76]. Our data on PA engagement do not paint an encouraging picture: more than 60% of people are well below the WHO recommended lower limits for physical exercise practice and only 22% practiced PA for more than 150 min throughout the week. In line with most literature data [26], we confirm that higher levels of PA correlate positively with better scores for mental health assessment indexes, including the 9-item SWB and its positive domain. Correspondingly, those who did not practice PA were about 50% more likely to be depressed and anxious and to suffer a worsened state of psychological well-being.

Our results are in agreement with previous data from Fluetsch et al. [77] and Silva et al. [78], who also found an inverse relationship between PA and mental health for those who reported being insufficiently active.

To support these observational data, a meta-analysis of studies that evaluated PA based interventions showed significant improvement in depressive symptoms in healthy adults, particularly when PA prescription included flexibility, resistance, and low-intensity exercise [79]. To further support, a meta-analysis including prospective studies with a follow-up of at least one year showed that higher levels of self-reported PA compared with lower levels of PA were associated with a lower risk of anxiety [80].

In recent years, the perception of lack of energy and fatigue has become so widespread and psychologically debilitating that it was recently reviewed as a major health problem. One in five adults worldwide reports persistent fatigue [81] and several studies have linked both feelings of low energy and high fatigue to a decreased likelihood of PA, low levels of personal and social activity [82], and increases in cardiovascular events and mortality [83]. In addition, both perceived energy and fatigue are recognized as prominent symptoms of various mental health conditions, including depression, anxiety, and insomnia [84]. More pertinently to the COVID-19 outbreak and associated lockdowns, it has been recently reported that pre-lockdown energy levels represented a negative predictor for quality of life in a cohort of physically active elderly people and that lower energy levels during lockdowns correlated with lower well-being perceptions [85].

The biological cause of fatigue is often difficult to determine, and consequently, the identification of effective treatment is challenging. Fortunately, recent data suggest that physical activity improves feelings of high energy and associates with low fatigue status [86]. Our data confirm previous observations regarding the distribution of these feelings among students and employees. In agreement with Morales-Vives et al., we observed that during lockdowns, older subjects feel better than younger counterparts [87] and in agreement with Puetz et al. [88] and Ellingson et al. [86] we also observed that energy scores positively correlate with PA practicing in both students and staff. However, when examining the relationship between PA and SWB, it is important to consider PA practice not only in terms of frequency and volume, but also in terms of “psychological climate”, including environmental and social aspects [32]. For this reason, we also examined the relationship between SWB and the frequency of leisure activities and time spent in contact with nature.

Evidence for a positive relationship between contact with the natural environment, health and well-being is getting increasingly clear [89,90], prompting a deeper understanding of the underlying exposure–response relationships [91]. Concordantly, PA that is carried out in nature is postulated to bring together the positive impacts of PA and contact with nature and even to have synergistic effects. In this context, many studies have highlighted the benefits of outdoor sports as going beyond being active in a non-natural (inside) environment [38]. However, beyond the health enhancing effects of PA and nature, outdoor sports are also associated with social benefits, including the intra- and interpersonal development, the active citizenship and the social connectedness and network [91]. We sought to assess these relationships by measuring direct exposure to the natural environment, defined as self-reported time spent recreating in nature in the last month, and self-reported health, SWB, distress and energy. After taking into account a series of covariates, we observed that both students and employees who spent more time in nature reported better general health and energy than those spending less time. On the other side, the beneficial effects in terms of SWB and distress were significant only for students, likely as a consequence of the better baseline mental health condition reported by staff members. Our data align with recent reports by White et al. and Colucci et al., who also highlighted a positive relationship between PA and mental health [85,92] and confirm that PA benefits may be mediated by contact with the natural environment [35]. One explanation for these results could be that time spent in nature simply promotes PA, which is the real factor in physical and mental health, rather than contact with nature per se. However, in line with Belanger et al. [35], mediation analysis confirmed a mediator role for time spent in nature, and interestingly, recognized in the quality of social relationships, and in the energy levels, further mediator roles in the relationship between PA and SWB.

Overall, our data furnish further support to the correlation between nature and related socio-ecological factors and physical and mental health [93]. They point out that people may benefit from living in a natural environment to maintain their health and well-being. This confirms and highlights the existence of a mutually beneficial relationship between humans and nature to promote a healthy lifestyle, including PA and nutrition [93]. Our findings may be of help for a better understanding of how socioecological and lifestyle factors affect health and well-being and will be of help to policymakers in taking more fitting decisions for the better management of the consequences of the COVID-19 pandemic, in particular for more fragile, younger individuals.

### Strengths and Limitations

This study has some potential limitations that have to be mentioned. Despite the large number of participants enrolled in this study, our sample was limited to highly-educated young students and middle-age adult employees. Therefore, it is possible that the reported levels of PA are much higher than that of the general population during the COVID-19 lockdown. Additionally, people who experienced some mental health difficulties might have been more inclined to participate in the study as they felt the topic was relevant. This may lead to an overestimation of depression and anxiety and underestimation of SWB. A further important limitation has to be recognized in the self-reported evaluation of PA practiced, which can lead to an overestimation of PA itself [94]. Our future research must necessarily take advantage of the use of validated questionnaires to measure levels of PA [95] or, even better, electronic devices to objectively measure levels of PA performed and possibly make a space-time geolocation to measure levels of contact with the natural environment. Again, the cross-sectional design of the study does not permit any conclusion concerning directionality in the relationship between PA levels and mental health and does not allow us to build any causal relationship, which means we cannot establish if low PA can affect depression, anxiety and stress symptoms as well as SWB, or if depression, anxiety and stress symptoms are the cause of low PA. The analysis of the biological mechanisms underlying the observed association may be of help to establish of the real causal association (Mill’s canons, [96]). Potential biological pathways involve the PA-mediated release of endorphins [97], the activation thermogenesis [98], as well as the release of neurotransmitters such as dopamine and serotonin [99,100]. Future longitudinal studies should further explore and confirm these mechanistic pathways. Finally, we do not have pre-pandemic mental health scores and, consequently, accurate pre-post analysis cannot be performed.

## 5. Conclusions

In conclusion, our data provide evidence for a positive association between PA and SWB. Results also show that PA exerts its beneficial effects if we spend more time in contact with the natural environment, this allowing an improvement in the quality of social relationships and in perceived energy levels. Our data also validate new “smarter” scores for a faster evaluation of SWB. These results provide important new benchmarks for a potentially easier identification of at-risk subjects, and therefore they may also be useful for tailoring psychological therapies. Deeper interventions aimed to promote and support outdoor activities are suggested in order to maintain and improve SWB.

## Figures and Tables

**Table 1:** Mental health screening tools, questions, and domains.

	What Measure		Original Items Number
DASS-21	Distress	Depression	3. I could not seem to experience any positive feeling at all;
			5. I found it difficult to work up the initiative to do things;
			10. I felt that I had nothing to look forward to;
			13. I felt down-hearted and blue;
			16. I was unable to become enthusiastic about anything
			17. I felt I was not worth much as a person;
			21. I felt that life was meaningless;
		Anxiety	2. I was aware of dryness of my mouth;
			4. I experienced breathing difficulty (e.g., excessively rapid breathing, breathlessness in the absence of physical exertion);
			7. I experienced trembling (e.g., in the hands)
			9. I was worried about situations in which I might panic and make a fool of myself;
			15. I felt I was close to panic;
			19. I was aware of the action of my heart in the absence of physical exertion (e.g., sense of heart rate increase, heart missing a beat);
			20. I felt scared without any good reason;
		Stress	1. I found it hard to wind down;
			6. I tended to over-react to situations;
			8. I felt that I was using a lot of nervous energy;
			11. I found myself getting agitated;
			12. I found it difficult to relax;
			14. I was intolerant of anything that kept me from getting on with what I was doing;
			18. I felt that I was rather touchy;
WHOQoL Brief	Psychological health (PH)		5. How much do you enjoy your life?;
			6. To what extent do you feel your life is meaningful?;
			7. How well can you concentrate?;
			11. Can you accept your physical appearance?;
			19. How satisfied are you with yourself?;
			26. How often do you have negative feelings such as bad mood, despair, anxiety, depression?;
	Social relationships (SR)		20. How satisfied are you with your personal relationships?;
			21. How satisfied are you with your sex life?;
			22. How satisfied are you with the support you get from your friends?
	General health (GH)		How satisfied are you with your health?
Subjective well-being (SWB)	9-item SWB	C1 (positive domain)	1.How satisfied are you with your life as a whole these days?
			2. To what extent do you feel that the things you do in life are worthwhile?;
			3. How happy did you feel during the last week?;
			8.Last week, how energetic did you normally feel in the middle of the day?
			9. Last week, how efficient did you normally feel in the middle of the day?
		C2 (negative domain)	4. How worried did you feel during the last week?
			5. Did you feel depressed during the last week?
			6. During last week, how often did you feel nervous and stressed?
			7. During last week, how often did you feel that you were unable to cope with all the thing you had
	Energy level		8.Last week, how energetic did you normally feel in the middle of the day?
			9. Last week, how efficient did you normally feel in the middle of the day?
			10. Last week, how tired did you normally feel in the middle of the day?

**Table 2:** Demographic characteristics and body health status (BMI and diagnosed pathology) in the students and staff sample subpopulations.

	Students	Staff	*p*-Value
**N (%)**	939	238	**<0.001**
**Sex distribution, N (%):**			
**Male**	235 (25.0)	109 (45.8)	
**Female**	704 (75.0)	129 (54.2)	
**Age distribution (years), N (%)**			**<0.001**
**18–24 y**	586 (62.4)	0 (0)	
**25–34 y**	274 (29.2)	8 (3.4)	
**35–44 y**	47 (5.0)	38 (16.0)	
**45–54 y**	26 (2.8)	96 (40.3)	
**55–64 y**	5 (0.5)	86 (36.1)	
**>65 y**	1 (0.1)	10 (4.2)	
**Marital status, N (%)**			**<0.001**
**Single**	676 (71.9)	58 (24.3)	
**Married or analogous relationship**	263 (28.0)	180 (75.6)	
**Having children N (%)**			**<0.001**
**No**	886 (94.4)	75 (31.5)	
**Yes**	53 (5.6)	163 (68.5)	
**Cohabitants**			**<0.001**
**Median (IQR)**	4.00 (1.00)	3.00 (2.00)	
**Mean ± SD**	3.69 ± 0.99	2.92 ± 1.17	
**Family income N (%)**			**<0.001**
**<500€**	51 (5.4)	0 (0)	
**500–1000€**	143 (15.2)	1 (0.4)	
**1000–1500€**	272 (29.0)	28 (11.8)	
**1500–2000€**	215 (22.9)	45 (18.9)	
**2000–2500€**	143 (15.2)	44 (18.5)	
**>2500€**	115 (12.2)	120 (50.4)	
**BMI (Kg/m^2^)**			**<0.001**
**Median (IQR)**	22.48 (5.00)	24.22 (4.00)	
**Mean ± SD**	23.14 ± 4.21	24.88 ± 4.00	
**BMI distribution ^1^, N (%)**			**0.002**
**Underweight**	72 (7.7)	1 (0.4)	
**Normal weight**	633 (67.4)	137 (57.6)	
**Overweight**	183 (19.5)	77 (32.4)	
**Obese**	51 (5.4)	23 (9.7)	
**Diagnosed pathologies, N (%)**			**0.002**
**No**	816 (86.9)	188 (79.0)	
**Yes**	123 (13.1)	50 (21.0)	

^1^ Categories of BMI according to the World Health Organization (WHO) [55] underweight < 18.5 kg/m^2^; normal weight ≥ 18.5–24.9 kg/m^2^; overweight 25.0–29.9 kg/m^2^; obesity ≥ 30.0 kg/m^2^; N = Sample size; IQR= interquartile range; SD = standard deviation. Mann–Whitney U tests were used to assess differences for scale variables; Chi-squared tests were used for nominal and ordinal variables (significant differences in bold when *p*-values < 0.05).

**Table 3:** DASS-21 scores of students and staff members according to the severity levels.

	Students		Staff		
Severity	(N)	%	N	%	*p* Value
*DASS—Depression*					
Normal (range: 0–9)	405	**43.2**	184	77.3	**<0.001**
Mild (range: 10–12)	126	13.4	23	9.7	0.127
Moderate (range: 13–20)	213	22.7	21	8.8	**<0.001**
Severe (range: 21–27)	95	10.1	5	2.1	**<0.001**
Extremely severe (range: 28–42)	100	10.6	5	2.1	**<0.001**
*DASS—Anxiety*					
Normal (range: 0–6)	571	**60.8**	206	86.7	**<0.001**
Mild (range: 7–9)	78	8.3	12	5	0.09
Moderate (range: 10–14)	152	16.2	13	5.5	**<0.001**
Severe (range: 15–19)	58	6.2	3	1.3	**0.001**
Extremely severe (range: 20–42)	80	8.5	4	1.7	**<0.001**
*DASS—Stress*					
Normal (range: 0–10)	469	49.9	187	78.6	**<0.001**
Mild (range: 11–18)	64	6.8	14	5.9	0.605
Moderate (range: 19–26)	215	23	23	9.7	**<0.001**
Severe (range: 27–34)	128	13.6	12	5	**<0.001**
Extremely severe (range: 35–42)	63	6.7	2	0.8	**<0.001**

N = Sample size. Fisher’s exact test was used to assess differences between students and employees (significant differences in bold when *p*-values < 0.05).

**Table 4:** 9-item SWB index and subdomain validation.

N ^1^ (Valid Population)	Comparison Tool	Correlation ^2^ (r, *p* Value)	ICC ^3^ (95% CI, *p* Value)
9-item SWB index			
Students (939)	WHOQoL psychological health domain	0.762, <0.001 Strong positive correlation	0.713 (0.673, 0.747; <0.001) Moderate
	DASS-21 distress	−0.801, <0.001 Strong negative correlation	not calculable
Staff (238)	WHOQoL psychological health domain	0.734, <0.001 Strong positive correlation	0.678 (0.585, 0.751; <0.001) Moderate
	DASS-21 distress	−0.784, <0.001 Strong negative correlation	not calculable
C1 (positive)			
Students (939)	WHOQoL psychological health domain	0.753, <0.001 Strong positive correlation	0.715 (0.676, 0.750; <0.001) Moderate
Staff (238)	WHOQoL psychological health domain	0.712, <0.001 Strong positive correlation	0.695 (0.606, 0.764; <0.001) Moderate
C2 (negative)			
Students (939)	DASS-21 distress	0.740, <0.001 Strong positive correlation	0.557 (0.496, 0.610; <0.001) Moderate
	DASS-21 depression	0.663, <0.001 Moderate positive correlation	0.586 (0.529, 0.636; <0.001) Moderate
	DASS-21 stress	0.745, <0.001 Strong positive correlation	0.620 (0.568, 0.666; <0.001) Moderate
	DASS-21 anxiety	0.543, <0.001 Moderate positive correlation	0.420 (0.341, 0.490; <0.001) Poor
Staff (238)	DASS-21 distress	0.762, <0.001 Strong positive correlation	0.442 (0.279, 0.568; <0.001) Poor
	DASS-21 depression	0.676, <0.001 Moderate positive correlation	0.463 (0.307, 0.584; <0.001) Poor
	DASS-21 stress	0.760, <0.001 Strong positive correlation	0.551 (0.421, 0.652; <0.001) Moderate
	DASS-21 anxiety	0.524, <0.001 Moderate positive correlation	0.265 (0.052, 0.431; 0.009) Poor

^1^ N (valid population used in the analyses). ^2^ Pearson correlation between 9-item SWB and WHOQoL PW and DASS-21. ^3^ ICC: Intra-class Correlation Coefficient between 9-item SWB and WHOQoL PW and DASS-21 using the two-way mixed model and consistence. Bilateral significance considered for *p*-value < 0.05.

**Table 5:** PA and recreational activities in students and employees.

	Students	Staff	*p*-Value
***Sport practising, N (%)***			**0.003**
**Never**	270 (28.8)	97 (40.8)	
**Occasionally**	297 (31.6)	57 (23.9)	
**Regularly (<150 min per week)**	158 (16.8)	31 (13.0)	
**Regularly (>150 min per week)**	214 (22.8)	53 (22.3)	
***Leisure activity, N (%)***			**0.006**
**Activities that do not require physical activity**	301 (32.1)	62 (26.1)	
**Relaxing activities sometimes per week**	311 (33.1)	105 (44.1)	
**Sport or intense physical activity**	327 (34.8)	71 (29.8)	
***Time spent in nature, N (%)***			0.06
**Never**	131 (14.0)	34 (14.2)	
**Occasionally**	276 (29.4)	48 (20.2)	
**Sometimes**	290 (30.9)	84 (35.3)	
**Frequently**	171 (18.2)	54 (22.7)	
**Almost all the time**	71 (7.6)	18 (7.6)	

N = Sample size; Chi-squared tests were used to assess differences between students and staff (significant differences in bold when *p*-values < 0.05).

**Table 6:** Distribution of mental health scores relative to sport and time spent in nature.

	Sport Practice				Physical Activity in Leisure Time				Time Spent in Nature			
	Low	Moderate/High	*p* Value	Effect Size (r)	Low	Moderate/High	*p* Value	Effect Size (r)	Low	Moderate/High	*p* Value	Effect Size (r)
Students												
***DASS—Depression***												
N (%) Mean ± SD	567 (60.4) 1.00 ± 0.73	372 (39.6) 0.81 ± 0.69	**<0.001**	0.14 Small	612 (65.2) 0.98 ± 0.73	327 (34.8) 0.82 ± 0.70	**0.001**	0.12 Small	407 (43.3) 1.04 ± 0.74	532 (56.7) 0.84 ± 0.71	**<0.001**	0.14 Small
***DASS—Anxiety***												
N (%) Mean ± SD	567 (60.4) 0.60 ± 0.57	372 (39.6) 0.42 ± 0.47	**<0.001**	0.16 Small	612 (65.2) 0.59 ± 0.57	327 (34.8) 0.41 ± 0.46	**<0.001**	0.16 Small	407 (43.3) 0.63 ± 0.58	532 (56.7) 0.45 ± 0.49	**<0.001**	0.17 Small
***DASS—Stress***												
N (%) Mean ± SD	567 (60.4) 1.24 ± 0.70	372 (39.6) 1.07 ± 0.67	**<0.001**	0.12 Small	612 (65.2) 1.23 ± 0.70	327 (34.8) 1.08 ± 0.68	**0.003**	0.10 Small	407 (43.3) 1.32 ± 0.68	532 (56.7) 1.07 ± 0.68	**<0.001**	0.18 Small
***9-item SWB index***												
N (%) Mean ± SD	567 (60.4) 5.45 ± 1.53	372 (39.6) 6.01 ± 1.52	**<0.001**	0.19 Small	612 (65.2) 5.53 ± 1.54	327 (34.8) 5.93 ± 1.52	**<0.001**	0.14 Small	407 (43.3) 5.34 ± 1.52	532 (56.7) 5.93 ± 1.52	**<0.001**	0.19 Small
Staff												
***DASS—Depression***												
N (%) Mean ± SD	154 (64.7) 0.45 ± 0.48	84 (35.3) 0.37 ± 0.49	0.282	0.07 NS	167 (70.2) 0.46 ± 0.52	71 (29.8) 0.32 ± 0.36	**0.014**	0.12 Small	82 (34.5) 0.49 ± 0.53	156 (65.5) 0.38 ± 0.45	0.119	0.11 NS
***DASS—Anxiety***												
N (%) Mean ± SD	154 (64.7) 0.24 ± 0.29	84 (35.3) 0.23 ± 0.33	0.797	0.04 NS	167 (70.2) 0.26 ± 0.32	71 (29.8) 0.19 ± 0.27	0.089	0.10 NS	82 (34.5) 0.28 ± 0.36	156 (65.5) 0.22 ± 0.27	0.161	0.007 NS
***DASS—Stress***												
N (%) Mean ± SD	154 (64.7) 0.75 ± 0.55	84 (35.3) 0.69 ± 0.54	0.449	0.05 NS	167 (70.2) 0.74 ± 0.56	71 (29.8) 0.71 ± 0.53	0.685	0.002 NS	82 (34.5) 0.82 ± 0.57	156 (65.5) 0.68 ± 0.54	0.068	0.12 NS
***9-item SWB index***												
N (%) Mean ± SD	154 (64.7) 6.60 ± 1.35	84 (35.3) 7.03 ± 1.49	**0.031**	0.14 Small	167 (70.2) 6.62 ± 1.43	71 (29.8) 7.06 ± 1.33	**0.023**	0.13 Small	82 (34.5) 6.51 ± 1.33	156 (65.5) 6.88 ± 1.44	0.055	0.10 NS

N = Sample size; SD = standard deviation. Mann–Whitney U tests were used to assess differences between students and employees (significant differences in bold when *p*-values < 0.05).

**Table 7:** Correlations between sport practicing, leisure activities, time spent in nature and the self-perceived psychophysical well-being and health.

Parameters	Sport Practice (Spearman ρ/*p* Values) ^(1)^		PA in Leisure Time * (Spearman ρ/*p* Values) ^(1)^		Time Spent in Nature (Spearman ρ/*p* Values) ^(1)^	
	Students	Staff	Students	Staff	Students	Staff
**SWB**						
9-item SWB	**0.102/0.002**	**0.135/0.041**	**0.083/0.012**	**0.147/0.026**	**0.163/<0.001**	0.097/0.143
C1 (positive)	**0.133/<0.001**	0.116/0.080	**0.115/<0.001**	0.086/0.191	**0.163/<0.001**	0.98/0.138
C2 (negative)	−0.045/0.172	−0.102/0.125	−0.025/0.443	**−0.130/0.049**	**−0.121/<0.001**	−0.075/0.257
Energy level	**0.187/<0.001**	**0.184/0.005**	**0.194/<0.001**	**0.170/0.010**	**0.187/<0.001**	**0.198/0.003**
**WHOQoL-Brief**						
Psychological health	**0.072/0.028**	**0.243/<0.001**	0.049/0.131	**0.208/0.001**	**0.106/0.001**	**0.142/0.032**
General health	**0.374/<0.001**	**0.396/<0.001**	**0.351/<0.001**	**0.360/<0.000**	**0.160/<0.001**	**0.209/0.001**
**DASS-21**						
Stress	−0.039/0.235	−0.082/0.218	−0.051/0.118	−0.036/0.592	**−0.116/<0.001**	−0.096/0.148
Anxiety	**−0.111/<0.001**	−0.075/0.257	**−0.119/<0.001**	−0.061/0.356	**−0.134/<0.001**	−0.036/0.586
Depression	**−0.067/0.042**	−0.080/0.228	**−0.070/0.036**	−0.069/0.297	**0.097/0.003**	−0.124/0.060
Distress	**−0.081/0.014**	−0.097/0.143	**−0.087/0.008**	−0.061/0.355	**−0.134/<0.001**	−0.107/0.107

^(1)^ Spearman partial correlation was used to assess the association between mental health indexes and Sport Practice, PA in Leisure Time, Time Spent in Nature. Correlations were controlled for sex, age, pathology, marital status, family income, WHOQoL-Brief social relationship quality score, smoking habits, sleep habits. Significant differences are shown in bold when *p*-values < 0.05). * leisure activity encompasses both the practice of sports in leisure time (higher score) and activities that do not require physical exertion (lower score).

**Table 8:** Mediation values coefficients.

Path		β	SE	*p*	*95%CI*	
					*Boot_LLCI*	*Boot_ULCI*
Sport practice→	9-item SWB	0.045	0.26	0.083	−0.094	0.007
	Time spent in nature	0.224	0.029	**<0.000**	0.168	0.281
	Quality of social relationship	0.073	0.021	**<0.001**	0.031	0.115
	Energy levels	0.205	0.039	**<0.000**	0.128	0.282
Time spent in nature→	9-item SWB	0.042	0.026	0.101	−0.007	0.089
	Quality of social relationship	0.086	0.021	**<0.001**	0.044	0.128
	Energy levels	0.210	0.038	**<0.001**	0.133	0.286
Quality of social relationship→	9-item SWB	0.402	0.037	**<0.001**	0.324	0.481
	Energy levels	0.571	0.054	**<0.001**	0.455	0.689
Energy levels→	9-item SWB	0.635	0.019	**<0.001**	0.595	0.673
**Indirect paths**						
Sport practice→Time spent in nature→9-item SWB		0.007	0.004	/	−0.001	0.015
Sport practice→Quality of social relationship→9-item SWB		0.021	0.006	/	0.009	0.034
Sport practice→Energy levels→9-item SWB		0.093	0.018	/	0.058	0.129
Sport practice→Time spent in nature→quality of social relationship→9-item SWB		0.006	0.002	/	0.003	0.009
Sport practice→Time spent in nature→Energy levels→9-item SWB		0.021	0.005	/	0.013	0.032
Sport practice→Quality of social relationship→Energy levels→9-item SWB		0.019	0.006	/	0.008	0.031
Sport practice→Time spent in nature→Quality of social relationship→Energy levels→9-item SWB		0.005	0.001	/	0.002	0.008

## Data Availability

Results attained in this study are included in the manuscript. Individual data are not publicly available due to ethical restrictions.

## Supplementary Materials

The following supporting information can be downloaded at: https://www.mdpi.com/article/10.3390/ijerph19084787/s1, Table S1: Correlation of 9-item SWB and energy levels with sport practice, physical activity in leisure time and time spent in nature.
