# Supplementary material for: Assessment of Subjective Well-Being in a Cohort of University Students and Staff Members: Association with Physical Activity and Outdoor Leisure Time during the COVID-19 Pandemic
Source: Int J Environ Res Public Health. 2022 Apr 14;19(8):4787. doi: 10.3390/ijerph19084787 (PMC9025284; doi:10.3390/ijerph19084787)
Supplement: Supplementary file 1 [file ijerph-19-04787-s001.zip › ijerph-1600029-supplementary.pdf]

Supplementary Table S1. Correlation of 9-item SWB and energy levels with sport practice, physical activity in leisure time and time spent in nature.

	Sport practice	Physical activity in leisure time	Time spent in nature
9-item SWB	0.149***	0.160***	0.225***
Energy levels	0.222***	0.236***	0.243***

Pearson and Spearman correlation analyses were performed to examine the correlations of 9-item SWB and energy levels with sport practice, PA in leisure time, and time spent in nature. Note: *** $p < 0.001$.
